# Reviewer acknowledgment 2015

**DOI:** 10.1186/s12959-016-0079-z

**Published:** 2016-02-24

**Authors:** Hugo ten Cate, Yukio Ozaki

**Affiliations:** Academic Hospital Maastricht, Maastricht, The Netherlands; University of Yamanashi, Yamanashi, Japan

## Abstract

The Editors of *Thrombosis Journal* would like to thank all of our reviewers who have contributed to the journal in Volume 13 (2015) and whose valuable support is fundamental to its success.

**Robert Ariens**

UK

**Juan Badimon**

USA

**Erik Beckers**

Netherlands

**Lars Borris**

Denmark

**Annemieke Bouman**

Netherlands

**Suzanne Cannegieter**

Netherlands

**Mario M D'Elios**

Italy

**Matteo Nicola Dario Di Minno**

Italy

**Marcello Di Nisio**

Italy

**Jonathan Douxfils**

Belgium

**William Fisher**

Canada

**Satoshi Fujii**

Japan

**Alexander Gallus**

Australia

**Esteban Gandara**

Canada

**Hadi Goubran Messiha**

Canada

**José Govers-Riemslag**

Netherlands

**Nil Guler**

Turkey

**Karly Hamulyak**

Netherlands

**Yvonne Henskens**

Netherlands

**Masahiro Ieko**

Japan

**David Jiménez**

Spain

**Frederikus Klok**

Netherlands

**Takao Kobayashi**

Japan

**Amartya Kundu**

USA

**Seiji Madoiwa**

Japan

**Harry Magnani**

Netherlands

**Karina Meijer**

Netherlands

**Marco Moia**

Italy

**Mashio Nakamura**

Japan

**Marisa Ninivaggi**

Netherlands

**Michael Nurmohamed**

Netherlands

**Toshiyuki Ojima**

Japan

**Marina Panova-Noeva**

Germany

**Ron Pisters**

Netherlands

**Domenico Prisco**

Italy

**Jürgen Prochaska**

Germany

**Tomohiro Sakamoto**

Japan

**Masato Sakon**

Japan

**Carlos Santos-gallego**

USA

**Nobuyuki Sato**

Japan

**Craig Seaman**

USA

**Toshiro Shinke**

Japan

**Tomonori Sugiura**

Japan

**Hiroyuki Sumi**

Japan

**Hideyuki Takedani**

Japan

**Hiroki Tanaka**

Japan

**Arina Ten Cate-Hoek**

Netherlands

**Kazuo Umemura**

Japan

**Naoki Unno**

Japan

**Tom van der Hulle**

Netherlands

**Nick Van Es**

Netherlands

**Minka Vries**

Netherlands

**Jeanine Walenga**

USA

**Mark Walsh**

USA

**Theodore Warkentin**

Canada

**Siegfried Wieshammer**

Germany

**Kristien Winckers**

Netherlands

**Yi Wu**

USA

**Aizhen Yang**

China

**Urooj Zafar**

USA

